# Induction of immunogenic cell death by active components of natural products reshaping the tumor microenvironment for enhanced antitumor immunity

**DOI:** 10.3389/fphar.2026.1745590

**Published:** 2026-01-27

**Authors:** Xiaojie Chen, Haojia Li, Jianfei Chen, Boyang Hou, Zhaokun Tian, Wenyan Qiu, Zhiyong Yu

**Affiliations:** 1 The First Clinical Medical College, Shandong University of Traditional Chinese Medicine, Jinan, Shandong, China; 2 Shandong Cancer Hospital and Institute, Shandong First Medical University and Shandong Academy of Medical Sciences, Shandong Cancer Hospital and Institute, Jinan, Shandong, China; 3 School of Traditional Chinese Medicine, Binzhou Medical College, Yantai, Shandong, China

**Keywords:** cancer immunotherapy, damage-associated molecular patterns, immunogenic cell death, nanodelivery, natural products, tumor microenvironment

## Abstract

**Background:**

The immunosuppressive tumor microenvironment (TME) is a principal factor limiting the success of current cancer immunotherapies. Immunogenic cell death (ICD), a process whereby dying tumor cells elicit an adaptive immune response through the emission of damage-associated molecular patterns (DAMPs), offers a promising strategy to counteract this limitation. Natural products (NPs) constitute a valuable reservoir of compounds capable of triggering ICD.

**Purpose:**

This review aims to provide a comprehensive overview of ICD inducers derived from NPs, detail their molecular mechanisms of action, and explore their capacity to remodel the immunosuppressive TME.

**Methods:**

We performed a comprehensive literature search in relevant electronic databases to identify studies describing NPs-based components that stimulate characteristic ICD markers, such as surface exposure of calreticulin (CRT), and extracellular release of ATP and high mobility group box 1 (HMGB1).

**Results:**

Active components of NPs were demonstrated to initiate ICD largely via the induction of endoplasmic reticulum (ER) stress and reactive oxygen species (ROS). The consequent emission of DAMPs facilitates dendritic cell-mediated phagocytosis of tumor antigens and the priming of cytotoxic T lymphocytes. Additionally, the inflammatory milieu generated by NPs-induced ICD reprograms the TME by promoting the repolarization of macrophages to an immunostimulatory M1 phenotype and inhibiting the suppressive functions of myeloid-derived suppressor cells (MDSCs).

**Conclusion:**

Inducing ICD with NPs is a viable therapeutic strategy to potentiate anti-tumor immunity. The convergence of NPs-based ICD inducers with nanotechnology-based delivery systems offers a robust platform for the development of innovative combination regimens aimed at improving patient outcomes.

## 1 Introduction

The tumor microenvironment (TME) is a term that collectively refers to the special environment in which tumor cells live, composed of various types of cells within the tumor, tumor blood vessels, secreted factors, and the extracellular matrix, critically influences cancer progression and therapeutic response (Xiao and Yu, 2021). Immunotherapy for tumors has become an important treatment method and markedly advanced oncology (Yang et al., 2023). Immune checkpoint inhibitors (ICIs) targeting the PD-1/PD-L1 axis have demonstrated substantial clinical efficacy (Doroshow et al., 2021; Yap et al., 2023; Ricci et al., 2025). However, a key barrier to effective treatment is an immunosuppressive TME, which is often infiltrated by suppressive immune cells such as myeloid-derived suppressor cells (MDSCs) and regulatory T cells (Tregs), that inhibit the activity of cytotoxic T lymphocytes (CTLs) (Baldominos et al., 2022; Wang Z. et al., 2021). Therapeutic resistance poses another severe challenge for a substantial portion of patients, and is usually associated with CTLs deficiency and the immunosuppressive microenvironment mentioned above, particularly common in “cold tumors” that exhibit lower immunogenic activity (Bagchi et al., 2021; Wang Z. et al., 2023; Liu et al., 2022). Recent clinical evidence and consensus analyses have further reinforced this perspective. For instance, a 2025 meta-analysis of nivolumab plus ipilimumab in non-squamous non-small cell lung cancer demonstrated that patients with high tumor proportion score (TPS) had significantly longer progression-free survival (PFS) than those with low TPS (mPFS:12.4 vs. 6.6 months), underscoring the fundamental barrier posed by the immunosuppressive TME (Miyakoshi et al., 2025). Consequently, developing innovative strategies to convert immunologically “cold tumors” into “hot tumors” represents an urgent medical need.

A promising approach to achieving this conversion is through the induction of immunogenic cell death (ICD) (Galluzzi et al., 2024; Kroemer et al., 2022; Asadzadeh et al., 2020). Unlike tolerogenic apoptosis, ICD is a functionally distinct form of regulated cell death that triggers an adaptive immune response against antigens derived from dying tumor cells (Li Z. et al., 2022; Yu et al., 2023). The immunogenicity of ICD is defined by the release of damage-associated molecular patterns (DAMPs), which function as danger signals to activate the immune system. This process involves the spatiotemporally defined exposure of calreticulin (CRT) on the plasma membrane, acting as an “eat-me” signal for dendritic cells (DCs) (Galluzzi et al., 2020; Elliott et al., 2009). The subsequent extracellular release of adenosine triphosphate (ATP) serves as a chemotactic factor for antigen-presenting cells (APC) and activates the NOD-like receptor thermal protein domain associated protein 3 (NLRP3) inflammasome via the P2X7 receptor (Gombault et al., 2012). Finally, the release of high mobility group box 1 (HMGB1) protein promotes DCs maturation and antigen processing by engaging Toll-like receptor 4 (TLR4) (Özbay Kurt et al., 2024). Ultimately, it promotes the efficient generation of CD8^+^ T cells, initiating a potent and sustained antitumor immunity. Most critically, ICD induction is equivalent to an “*in situ* vaccination”, activating and recruiting T cells within the tumor that can recognize and attack it, creating the necessary conditions for ICIs to work, thereby overcoming primary resistance (Wu Y. et al., 2023).

Accumulating evidence indicates that numerous suggests that a multitude of active constituents sourced from Natural Products (NPs), such as terpenoids, flavonoids, and quinones, exhibit potent antitumor capabilities (Nan Y. et al., 2022). Beyond direct cytotoxicity, these NPs are being increasingly acknowledged for their capacity to regulate immune responses and the TME (Wang K. et al., 2021; Su et al., 2025; Liu QP. et al., 2023). Notably, certain NPs components can stimulate the release of DAMPs, which is a characteristic feature of ICD (Chen YY. et al., 2024; Fan et al., 2021; Han et al., 2023). However, to unequivocally classify a compound as a true ICD inducer, it must be validated through a rigorous, multistep framework. First, there must be molecular evidence of significant DAMP exposure; next, functional evidence of dendritic cell activation and antigen cross-presentation to CD8^+^ T cells; and finally, *in vivo* evidence from vaccine re-challenge assays demonstrating that it induces protective, antigen-specific immunity. Based on this rigorous framework, NPs discussed herein are categorized as canonical “ICD inducers” only when supported by the complete evidence chain, or as “ICD-like modulators” when evidence is partial.

Gene mutations in tumor cells may lead to the loss of certain antigens, which is one of the important reasons for the development of resistance to immunotherapy (O'Donnell et al., 2019). Notably, ICD has the capacity to activates T-cells against a wide array of tumor antigens. Even when some tumor cells lose a specific antigen through mutation, the immune system can continue to target the tumor via alternative antigens, thereby substantially raising the threshold for immune escape (Gedik et al., 2024). ICD has the capacity to activate T - cells against a wide array of tumor antigens. Tumor cells are prone to genetic mutations, which may lead to the loss of a particular antigen (Han et al., 2021). However, thanks to ICD, even when some tumor cells undergo such mutations and lose a specific antigen, the immune system can still effectively target the tumor by recognizing alternative antigens. This mechanism helps maintain the therapeutic effect and reduces the occurrence of immune escape (Mei et al., 2024; Yu et al., 2024). Endoplasmic reticulum (ER) stress and reactive oxygen species (ROS) bursts during ICD can promote the modification of tumor antigens and enhance the efficiency of antigen presentation by DCs, thereby generating more specific effector T cells (Yu et al., 2023). On the other hand, antigens released by dying cells can be captured by DCs and presented in a more persistent form, providing continuous antigen stimulation for the generation and maintenance of memory T cells (Garg and Agostinis, 2017).

ICIs have transformed cancer therapy, but their optimal use is still constrained by lack of response and toxicity (Goodman et al., 2023). In addition, many cytotoxic ICD inducers are associated with dose-limiting systemic toxicity (Huang et al., 2025). The core advantage of combining ICIs with ICD inducers lies in reducing the required doses of each through synergistic effects, thereby avoiding the dose-limiting toxicity issues of both, alleviating side effects and improving patient quality of life (Catanzaro et al., 2025). NPs are ideal candidates for ICD induction due to their diverse biological activities, low toxicity, and potential immunomodulatory abilities. NPs have made certain progress in inducing ICD and combination therapy, but their clinical translation still faces key challenges, mainly including poor water solubility, low bioavailability, and the resulting off-target toxicity (Jiang et al., 2025). Advances in nanotechnology offer promising strategies to overcome these barriers, enabling the enhanced and targeted delivery of NPs-based ICD inducers to tumor sites (Zhang et al., 2019; Chen et al., 2025; Liang et al., 2024).

This review aims to provide a comprehensive overview of research progress on ICD induction (including confirmed ICD inducers and ICD-like modulators) by active components derived from NPs. It will first delineate the key molecular pathways through which these compounds trigger ICD, and subsequently focus on their complex mechanisms and multifaceted roles in reprogramming the immunosuppressive tumor microenvironment. Finally, the review explores advanced nanodelivery strategies designed to overcome translational barriers and discusses future clinical translation pathways and central challenges.

## 2 Active components of NPs inducing ICD and their molecular mechanisms

**FIGURE 1:**
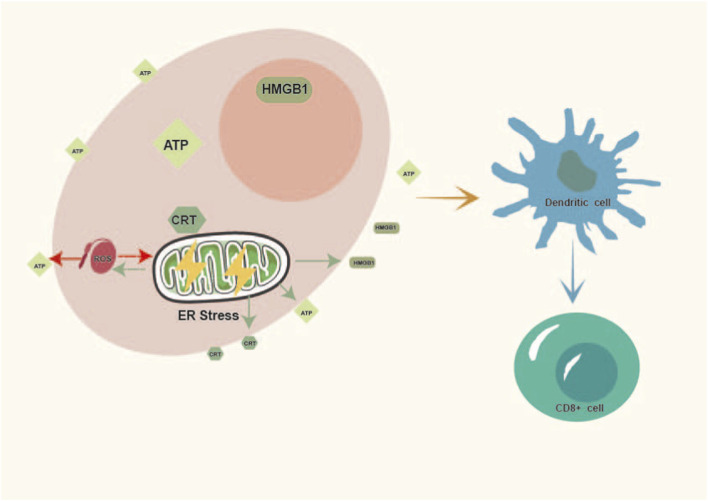
Core mechanisms of immunogenic cell stress and death. This schematic was created by the author based on the concepts and mechanisms described in this article. (CRT, calreticulin; ATP, adenosine triphosphate; HMGB1, high mobility group box 1; ROS, reactive oxygen species; ER stress, endoplasmic reticulum stress).

Substantial evidence has identified numerous bioactive components derived from NPs as potent inducers of ICD (Zhang et al., 2024; Yang LJ. et al., 2024). These structurally diverse compounds primarily induce the characteristic release of DAMPs by activating specific stress signaling pathways, with ER stress and ROS generation being central mechanisms, thereby triggering DC-mediated adaptive immune responses (Figure 1). To systematically summarize their functional characteristics, we list representative active components of NPs and key evidence of their induction of ICD (Table 1).

**TABLE 1:** Representative NPs-derived active components inducing immunogenic cell death.

Classification	Active component	Source herb	Key DAMPs	Primary molecular mechanisms	Key experimental models	Key findings
Terpenoids	Shikonin	Arnebia euchroma	ATP, HMGB1	ROS generation; ER stress	Mouse B16F10 melanoma cells	Shikonin induces ICD in tumor cells and enhances dendritic cell-based cancer vaccine
	Dihydroartemisinin	Artemisia argyi	CRT, ATP, HMGB1	ROS generation	Mouse Hepa1-6 or H22 cells; Murine hepatocellular carcinoma models	Dihydroartemisinin induces ICD and results in anti-cancer immune response through recruitment and activation of DCs and cytotoxic T lymphocytes
Flavonoids	Isoquercitrin	*Hypericum perforatum* L	CRT, ATP, HMGB1	ER stress	Human gastric cancer cell lines (HGC-27, AGS, MKN-45, and SNU-1)	Isoquercitrin modulates ICD in GC by promoting ER stress
	Astragalin	Astragalus membranaceus	CRT, ATP, HMGB1	ROS generation; ER stress	Human liver cancer cells (SMMC7721, Huh7, SK-HEP-1, HCCLM3, MHCC97H) and mouse liver cancer cells (H22, Hepa1-6)	Astragalin induces ICD in liver cancer by targeting NQO2, and increases DC maturation, enhances CD8^+^ T cell infiltration, and reduces regulatory T cells
Quinones	Emodin	Rheum palmatum	CRT, ATP, HMGB1	ER stress	Human NSCLC cells A549 and H1299	Emodin induces apoptosis of lung cancer cells through ER stress and the TRIB3 pathway
Polyphenolic	Resveratrol	Veratrum grandiflorum	CRT, ATP, HMGB1	ROS generation; ER stress	Mouse colon cancer CT26 cells	Polyphenolic induces ICD and DC maturation
Flavonoid glycoside	Afzelin	Houttuynia cordata thunb	HMGB1, ATP	ER stress; ROS generation; NQO2 pathways	Lung cancer cells A549 and H1299 cells	Afzelin induces immunogenic cell death against lung cancer by targeting NQO2

### 2.1 Terpenoids

Terpenoids represent a major class of NPs-based inducers of ICD. A representative example is Shikonin, a naphthoquinone pigment derived from the roots of *Arnebia euchroma*. The mechanism of Shikonin involves a massive generation of intracellular ROS, which acts as an initial trigger for severe ER stress (Chen et al., 2012). Additionally, Shikonin inhibits pyruvate kinase M2 (PKM2), thereby altering glucose metabolism and further contributing to its ICD-inducing activity (Chen et al., 2011). Across various cancer models, Shikonin-treated cells exhibit robust hallmarks of ICD, including CRT exposure, ATP secretion, and HMGB1 release, ultimately leading to DC-mediated T cell priming both *in vitro* and *in vivo* (Liang et al., 2024; Li et al., 2021; Feng et al., 2023). Dihydroartemisinin, a terpenoid isolated from Artemisia argyi, induces ICD in hepatocellular carcinoma (HCC), as confirmed by the release/surface exposure of DAMPs and protective vaccination activity *in vivo*. Mechanistically, DHA inhibits cyclin-dependent kinases (CDKs), leading to intracellular ROS accumulation and subsequent ICD. In Hepa1-6 and H22 tumor-bearing mouse models, DHA exhibited antitumor activity by increasing tumor-infiltrating CD8^+^ T cells, while also activating dendritic cells expressing MHC-II, CD80, and CD86 (Zhou et al., 2024).

### 2.2 Flavonoids

Flavonoids, traditionally recognized for their antioxidant activity, can paradoxically exert pro-oxidant effects within the high-stress microenvironment of tumor cells to initiate ICD (Slika et al., 2022). A representative example is Isoquercitrin, isolated from plants such as *Hypericum perforatumL*, is an effective ICD inducer that stimulates the extracellular release of characteristic surface CRT, ATP, and HMGB1 in human gastric cancer AGS cells. In addition, Isoquercitrin upregulates the intracellular expression of heat shock protein 70 (HSP70) and HSP90 in a dose-dependent manner and is associated with the induction of ER stress (Liu J. et al., 2023).

Astragalin (ASG), a bioactive compound derived from *Astragalus membranaceus*, induces mitochondrial-dependent apoptosis and triggers endoplasmic reticulum (ER) stress through binding to NAD(P)H dehydrogenase quinone 2 (NQO2). This process promotes ICD, as evidenced by the surface exposure of CRT, translocation of HMGB1 and release of ATP. Furthermore, in a mouse model of liver cancer, ASG enhanced tumor immunogenicity and reversed the immunosuppressive tumor microenvironment, supporting its potential as an immunomodulatory agent (Zheng et al., 2025).

### 2.3 Quinone

Quinone compounds often act as redox cyclers, generating substantial oxidative stress that makes them potent ICD-like inducers. Emodin, an anthraquinone from Rheum palmatum, is a prime example. It induces a massive ROS burst, severe ER stress, and intracellular Ca^2+^ overload, collectively leading to release of DAMPs (Su et al., 2017). Unfortunately, there is no further *in vivo* evidence from testing that emodin induces protective antigen-specific immunity.

### 2.4 Polyphenolic

Resveratrol is a polyphenolic compound extracted from natural plants that disrupts the redox homeostasis within cells, leading to the rapid accumulation of reactive oxygen species. At the same time, it disturbs calcium flux and protein folding in the endoplasmic reticulum, activating the unfolded protein response and triggering a series of signaling events, releasing key damage-associated molecular patterns. Moreover, Resveratrol converts dying cells into tumor antigens, promoting the maturation of dendritic cells (Li et al., 2025).

### 2.5 Flavonoid glycoside

Afzelin, derived from Houttuynia cordata Thunb, promotes CRT exposure and the extracellular release of HMGB1 and ATP. It induces ER stress and ICD by upregulating key UPR markers and downregulating NQO2, thereby modulating cell proliferation, apoptosis, and ER stress pathways (Xia et al., 2023). Unfortunately, neither provides *in vivo* evidence from vaccine rechallenge tests to prove that they induce protective antigen-specific immunity.

Despite structural diversity, NPs capable of inducing ICD commonly led to rapid intracellular accumulation of ROS, triggering severe ER stress and the UPR. This pathway serves as a master switch for the release of DAMPs. ER stress promotes CRT translocation to the cell membrane, while ROS bursts not only intensify ER stress but also stimulate ATP secretion and membrane permeabilization, facilitating passive HMGB1 release. Thus, the ER stress/ROS axis represents a universal mechanism essential for ICD induction by NPs. Specificity arises from distinct interactions with molecular targets, and some compounds, such as Dihydroartemisinin and Juglone, synergistically activate alternative death pathways like ferroptosis, promoting more extensive antigen release and stronger inflammatory signaling. This parallel activation can amplify antigen availability, thereby provoking a more sustained antitumor immune response. However, potential overlapping toxicities need to be considered when different cell death pathways are activated simultaneously. Although synergistically inducing ICD is beneficial for anti-tumor immunity, it may affect protective mechanisms in normal tissues that share common signaling, such as the ER stress/ROS axis. These non-tumor-specific effects may manifest as increased acute organ toxicity or chronic immunopathology, potentially influencing the dosing regimens necessary to achieve durable immune responses in clinical applications.

## 3 Reprogramming the TME by NPs-induced ICD

**FIGURE 2:**
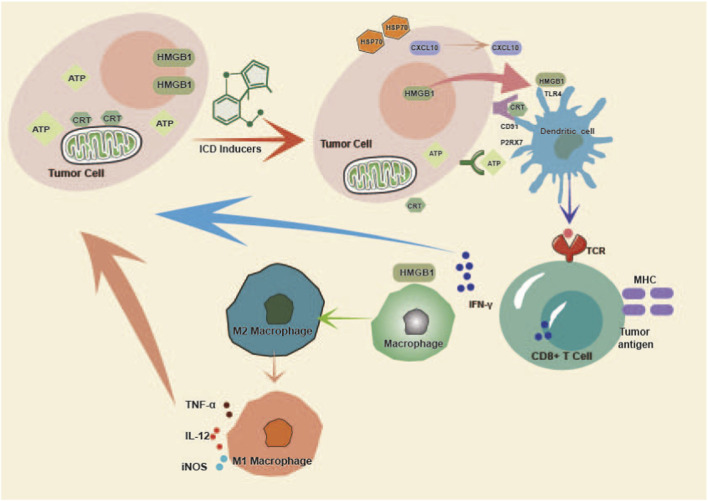
NPs-induced ICD converts tumors from an immune-cold state to a hot state through DAMP-mediated immune activation. (Tumor cells express and release DAMPs on their surface, which bind to pattern recognition receptors on DC cells, stimulating tumor antigen presentation, activating CD8 T cells to enhance anti-tumor immune responses, while also regulating the phenotype of tumor-associated macrophages by polarizing M2 macrophages into M1 macrophages, thereby remodeling the immunosuppressive tumor microenvironment. HSP70/90, heat shock protein 70/90; CXCL10, C-X-C motif chemokine 10; TLR4, Toll-like receptor 4; CD91 (official name: LRP1), LDL receptor-related protein 1; P2RX7, purinergic receptor P2X7; TCR, T-cell receptor; MHC, major histocompatibility complex; IFN γ, interferon γ; TNF-α, tumor necrosis factor-alpha; IL12, interleukin-12; iNOS: inducible nitric oxide synthase).

The induction of ICD extends beyond a cell-autonomous event to initiate profound reprogramming of the TME. By converting an immunosuppressive TME into an immunostimulatory TME, ICD triggers anti-tumor immune response. DAMPs released during ICD serve as critical danger signals that engage both innate and adaptive immunity (Figure 2). This section delineates how NPs-induced ICD reshapes the TME to drive a potent anti-tumor immune attack.

### 3.1 Activation of innate immunity: dendritic cells and macrophages

The ICD of tumor cells triggers the activation of antigen-presenting cells (APCs), with DCs playing a central role in bridging innate and adaptive immunity. Surface-exposed CRT acts as a potent “eat-me” signal by engaging the CD91 receptor on DCs, thereby promoting the phagocytosis of tumor-associated antigens (Liu et al., 2024; Luo et al., 2023). Extracellular ATP further stimulates the P2X7 receptor and NLRP3 inflammasome in DCs, leading to caspase-1-dependent secretion of interleukin-1β (IL-1β) and IL-18 (Sun et al., 2022; Moriya et al., 2022; Huang et al., 2024). Moreover, released HMGB1 further binds to Toll-like receptor 4 (TLR4) on DCs, upregulating major histocompatibility complex (MHC) I/II and co-stimulatory markers such as CD80/CD86, which collectively facilitate DC migration to draining lymph nodes and efficient antigen presentation to naïve T cells (Lau et al., 2020; Son et al., 2016). Consistent with this paradigm, tumor cells treated with Shikonin or Oridonin consistently induce potent DC activation and subsequent cross-priming of CTLs *in vivo* (Li J. et al., 2022).

The TME is frequently characterized by M2-polarized TAMs, which support tumor progression and immune evasion (Yang et al., 2025). The inflammatory milieu generated by NP-induced ICD can reprogram this phenotypic balance (Mao et al., 2022). Signals such as interferon-γ (IFN-γ) and DAMPs like HMGB1 can drive the repolarization of TAMs from an M2 toward an M1-like phenotype (Li et al., 2024; Zhao et al., 2021). M1 macrophages produce pro-inflammatory cytokines (e.g., TNF-α, IL-12), express inducible nitric oxide synthase (iNOS), and exhibit enhanced antigen-presenting capacity, thereby contributing directly to tumor cell killing and promoting Th1-type immune responses. The inflammatory signals elicited by NPs-induced ICD can shift this phenotypic balance. For instance, artesunate has been reported to inhibit M2 polarization and promote an M1-like macrophage phenotype, contributing to a more immunostimulatory TME (Hu et al., 2025).

### 3.2 Activation of adaptive immunity and modulation of suppressive cells

Effective antitumor immunity relies on the activation of CD8^+^ CTLs, which is initiated by DC-mediated antigen presentation. NPs capable of inducing ICD enhance both the priming and tumor infiltration of CTLs thereby fostering an immunologically “hot” tumor microenvironment (Gedik et al., 2024). ATP released from dying cells further supports CTL function within the metabolically challenging TME. For example, γ-mangostin promotes ICD and activates cyclic GMP-AMP synthase (cGAS) signaling, enhancing chemokine release and CD8^+^ T cell activation, leading to superior tumor control (Long et al., 2023). However, CTL activity is frequently restrained by immunosuppressive cell populations, notably MDSCs and regulatory Tregs. Inflammatory cytokines elicited during ICD, such as IL-1β and IFN-γ, can suppress MDSC differentiation and function (Feng et al., 2021). Furthermore, certain NPs, like Curcumin, have been reported to reduce the abundance or suppressive activity of MDSCs and Tregs, potentially facilitating more effective CTL-mediated tumor control (Wang T. et al., 2022).

### 3.3 Remodeling the tumor vasculature and stroma

Inflammatory signals elicited by ICD also promote remodeling of the non-cellular components of the TME (Prasad et al., 2023). Typically dysfunctional and poorly perfused, the tumor vasculature can undergo partial normalization in response to cytokines such as IFN-γ released during ICD, thereby improving blood flow and enhancing the delivery of both immune cells and therapeutic agents into the tumor core (Zheng et al., 2023; Wang et al., 2024a). Furthermore, cancer-associated fibroblasts (CAFs), which often contribute to immunosuppression and form a dense physical barrier, can be modulated under ICD-induced inflammatory conditions by downregulating the expression of TGF-β1, thereby inhibiting the phosphorylation and nuclear translocation of downstream Smad2/3 (Nan P. et al., 2022). In addition, activated CAFs rely on the PI3K/Akt pathway to maintain their proliferation and biosynthesis. ATP and ROS released during the ICD process can act as stress signals, inhibiting the PI3K/Akt activity in CAFs (Zhang et al., 2020). Under normal conditions, CAFs isolate CTLs at the periphery of the tumor nest by secreting CXCL12 and creating a physical barrier (Lafta et al., 2023). In a colon cancer model treated with a combination of curcumin and oxaliplatin, the mRNA expression and protein deposition of type I and type III collagen in tumor tissues were significantly reduced, the matrix structure has become loose (Zangui et al., 2019). Reduced CAF proliferation and extracellular matrix production may decrease stromal stiffness and facilitate improved T cell infiltration into tumor tissues (Cannone et al., 2022).

**FIGURE 3:**
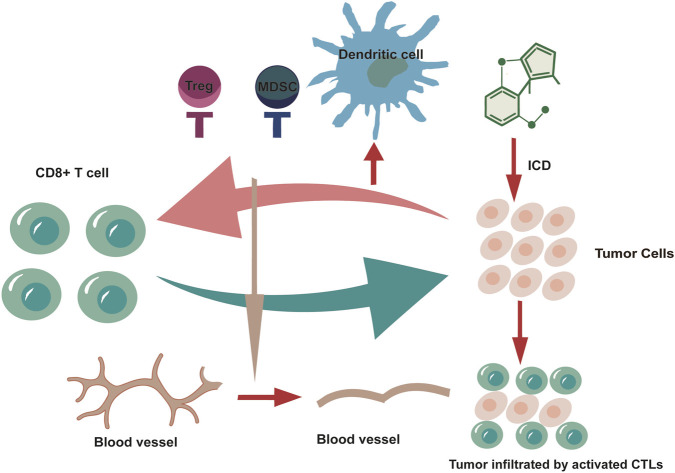
NP-induced ICD regulates the immune functions of CD8^+^ T cells and TAMs, forming a positive immune loop, while inhibiting immunosuppressive cells and the stromal barrier, creating conditions favorable for the activity and infiltration of immune cells. (Treg, Regulatory T cells; MDSC, Myeloid-derived suppressor cells; CTLs, Cytotoxic T lymphocytes).

In essence, NPs-induced ICD serves as a pivotal trigger for the reprogramming of the tumor TME. It initiates a self-propagating cycle of immune activation: DAMPs released from dying cells activate DCs, which in turn prime CTLs. The effector functions of these CTLs further potentiated by the repolarization of macrophages toward an anti-tumor phenotype and suppression of immunosuppressive cell populations (Han et al., 2022) (Figure 3). Collectively, NP-based ICD inducers operate beyond mere cytotoxic agents; they serve as potent immunomodulators capable of remodeling the immunosuppressive TME, offering a promising strategy for synergistic combination regimens (Mardi et al., 2022; Ren et al., 2025). However, a primary hurdle is the standardization and reproducibility of NP-derived compounds, given the natural variability in their sources and compositions, which complicates mechanistic validation and clinical batch production (Birer-Williams et al., 2020). Therefore, we propose that more complex experimental models are needed to integrate preclinical findings with human pathophysiology. For example, patient-derived tumor organoids can be co-cultured with peripheral blood mononuclear cells or autologous tumor-infiltrating lymphocytes. By simulating the patient-specific tumor microenvironment structure and cellular heterogeneity, this is used to assess whether NP compounds can remodel the TME and activate autologous immune cell responses in a personalized context. Alternatively, NPs therapy can be carried out in models where patient tumor tissues are transplanted into mice with humanized immune systems, combining *in vivo* imaging to observe the dynamic infiltration of immune cells, while performing spatial transcriptomics and mass cytometry analysis of tumor tissues at different time points. This can reveal the changes in immune phenotypes and signaling pathways in different regions of the tumor after NP treatment, directly verifying whether its multi-target effects exert a synergistic anti-tumor effect through the immune system.

## 4 Overcoming translational hurdles: combination strategies and nano-delivery

Beyond the challenge of standardization, the clinical translation of NP-based ICD inducers is further constrained by intrinsic pharmacological limitations (Kalachaveedu et al., 2023). Many promising NP compounds exhibit suboptimal pharmacokinetic properties, such as poor aqueous solubility, low oral bioavailability, and nonspecific tissue distribution, that severely restrict their effective delivery to tumor sites and attainment of therapeutic concentrations (Andreani et al., 2024). Comprehensive strategies are actively being explored, including rational combinations with chemotherapy and immunomodulators, as well as for targeted nanotechnology delivery to reprogram the immune environment.

### 4.1 Combinatorial therapies: synergizing with conventional and immunomodulatory agents

The combination of NPs-induced ICD with conventional cancer therapies represents a rational strategy to enhance antitumor efficacy and overcome therapeutic resistance (Table 2). Several standard therapies, including specific chemotherapeutic agents (e.g., anthracyclines, oxaliplatin) and radiotherapy, are established inducers of ICD (Sprooten et al., 2023; Giglio et al., 2018). The strategic co-administration of NPs-based ICD inducers with these treatments can amplify the immunogenic response. The synergy may arise from the complementary activation of distinct cell death and stress pathways (Hartleben et al., 2021; Nagaprashantha et al., 2012). For instance, a chemotherapeutic agent may cause DNA damage, alongside an NP component triggering severe ER stress, leading to more robust DAMP release than either agent alone (Wu PJ. et al., 2023). This approach may reduce the dosage of cytotoxic drugs, potentially alleviating side effects while maintaining or enhancing immunotherapeutic outcomes (Wang C. et al., 2024). Radiotherapy effectively induces localized tumor cell death but frequently lacks efficacy against distant metastases. Combining radiotherapy with systemically administered NPs-based ICD inducers may addresses this limitation (Sun et al., 2022; Liu T. et al., 2023). The NPs component can sensitize tumor cells to radiation-induced death by ensuring that radiation-induced cell death is potently immunogenic, thereby stimulating a systemic antitumor immune response that can target untreated lesions (Jafari et al., 2024).

**TABLE 2:** Strategies for enhancing the efficacy of NPs -induced ICD.

Strategy	Mechanism of enhancement	Representative example
Combination with chemo/Radiotherapy	Complementary activation of distinct cell death and stress pathways (e.g., DNA damage + ER stress), leading to amplified DAMP release	Pinellia pedatisecta schott lipid-soluble extract + cisplatin Chrysin + Radiation
Combination with ICIs	ICD “primes” the TME with T cells; ICB (anti-PD-1) prevents exhaustion of these activated T cells, synergizing to break immune tolerance	Camptothesome + PD-1 antibody
Nano-delivery (liposomes, polymers)	Improves solubility, stability, and circulation time of NPs compounds; enables passive targeting via the EPR effect	Liposomal curcumin
Active targeted Nanoparticles​	Surface ligands enable specific binding and uptake into tumor cells, increasing local drug concentration and ICD induction	A pH/glutathione (GSH) dual-responsive nano-herb delivery system for dihydroartemisinin
Stimuli-responsive nanosystems	Drug release is triggered specifically in the TME (by low pH, high ROS, enzymes), minimizing systemic toxicity and maximizing local efficacy	pH-sensitive polymeric nanoparticles for co-delivery of doxorubicin and curcumin

The combination of NP-induced ICD with ICB represents a powerful strategy to overcome therapeutic resistance (Wang Y. et al., 2024). While NPs-induced ICD primes the immune system by generating tumor-specific T cells, the immunosuppressive TME can inhibit these cells through checkpoints like PD-1/PD-L1 (Kepp et al., 2019). NPs-induced ICD acts as an ideal companion for ICB by converting cold tumors into hot tumors, thereby creating a microenvironment responsive to ICB. This “prime and release” strategy which involves first activating the immune response with ICD inducers and then sustaining it with ICB, represents a cornerstone of the next-generation immunotherapy (Wang Z. et al., 2022; Wang et al., 2024d). For example, ​Shikonin​ treatment has been shown to increase tumor-infiltrating CD8^+^ T cells and upregulate PD-L1 expression as an adaptive resistance mechanism. The subsequent administration of an anti-PD-1 antibody prevents T cell exhaustion, leading to dramatically enhanced tumor eradication and the establishment of long-term immune memory in preclinical models (Tan Y. et al., 2025). In addition, the inflammatory milieu generated by ICD may dynamically regulate emerging checkpoints like KLRG1 (Herndler-Brand et al., 2018). Consequently, monitoring these dynamic changes could provide predictive biomarker for assessing the efficacy of combination therapies utilizing NPs-derived ICD inducers together with ICB.

### 4.2 Nanotechnology-enabled delivery: precision targeting and enhanced immunogenicity

Nanotechnology provides promising approaches to overcome the physicochemical and pharmacokinetic limitations of many NPs components (Zhou et al., 2019; Jia et al., 2024). These platforms enable passive tumor targeting by leveraging the enhanced permeability and retention (EPR) effect, thereby promoting preferential accumulation within tumor tissues (Bagheri et al., 2022; Hao et al., 2015). Nanocarriers can also enable active targeting through surface functionalization with ligands, such as folate or RGD peptides that bind to receptors overexpressed on tumor cells. This strategy enhances specificity and reducing off-target effects (Kang et al., 2023; Yang C. et al., 2024). Moreover, nanocarriers serve as versatile platforms for co-delivery, allowing a single vehicle to encapsulate both an ICD-inducing NP and an immunomodulatory agent (e.g., an immune adjuvant or a TME modulator), This ensures simultaneous delivery to the same cellular location for a potent, localized combinatorial effect (Luo et al., 2022).

**FIGURE 4:**
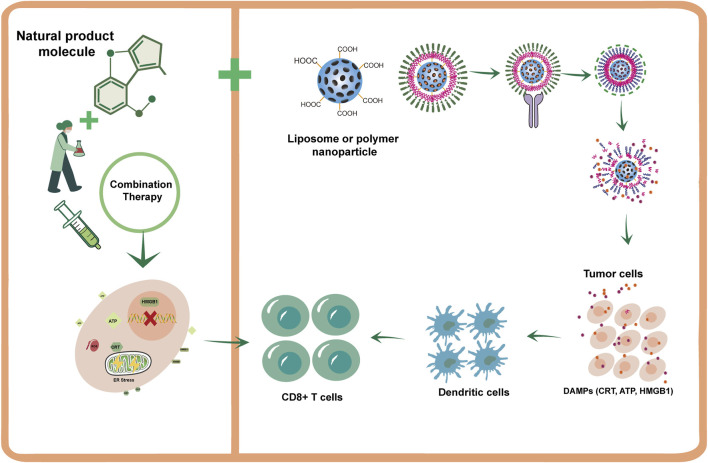
Synergistic strategies to enhance NPs-induced ICD. This schematic illustrates two key approaches: Xiao and Yu (2021) combining ICD-based NPs inducers with conventional therapies (chemotherapy/radiotherapy) or ICB to generate synergistic antitumor immune effects through complementary mechanisms; Yang et al. (2023) using nanotechnology-based delivery systems to improve the pharmacokinetics and efficacy of NP components, thereby achieving precise ICD induction.

Nanoparticles can also be engineered to be stimuli-responsive, enabling controlled drug release specifically in response to distinctive TME conditions such as low pH and elevated enzyme levels (Zhang et al., 2017). The rational design of these nanocarriers requires synergistic optimization of multiple parameters, including size/morphology (influencing biodistribution and the EPR effect), surface chemistry (affecting stability and biocompatibility), and targeting ligands (enabling cell-specific delivery), collectively aiming to achieve potent and localized ICD induction for enhanced immunotherapy (Yuan F. et al., 2025). A recent innovative approach involves coating nanoparticles with membranes derived from autologous cells, such as DCs, macrophages, or platelets. These biomimetic nanoparticles inherit surface proteins from their source cells, which can improve immune evasion and homologous targeting. This is analogous to prolonged ICD, enabling intelligent amplification of therapeutic signals (Wang T. et al., 2024). More specifically, DCs membrane-coated nanoparticle can not only deliver ICD inducers but also directly present tumor antigens to T cells, thereby integrating vaccine and immunogenic cell death functions into a single platform (Yang Y. et al., 2024; Srivastava et al., 2024) (Figure 4).

### 4.3 Potential applications of other delivery systems in delivering natural products

In addition to classic nanoparticles (e.g., polymeric nanospheres), there are many advanced delivery strategies that can be used to enhance the efficacy of ICD inducers (Table 3). Liposomes are one of the most extensively studied and clinically successfully translated nanocarrier delivery systems (Zou, 2023). Their advantage lies in being composed of a phospholipid bilayer made from natural components of cell membranes, capable of loading both hydrophilic drugs (encapsulated in the aqueous core) and hydrophobic drugs (embedded in the lipid bilayer). They can protect encapsulated drugs from degradation, prolong their half-life, and can be PEGylated (for long circulation) or actively targeted to tumor cells. However, premature drug leakage may occur. Doxorubicin liposomes were the first approved nanomedicine, and their ability to induce ICD has been widely studied. Research shows that compared with free doxorubicin, Doxil® can accumulate more effectively in tumors and activate anti-tumor immunity by inducing ICD (Wang H. et al., 2024; Wu et al., 2021).

**TABLE 3:** Comparison of different drug delivery systems for NPs-derived ICD inducers.

Delivery system	Core mechanism	Unique advantage	Main limitations	Clinical translation stage
Liposome ​	Hydrophilic drugs are encapsulated in the aqueous core, hydrophobic drugs are embedded in the phospholipid bilayer, and tumors are targeted through the EPR effect	High biocompatibility, Flexible drug loading, Mature technology, Long circulation	Drug leakage, low encapsulation efficiency, challenges in large-scale production	Doxil is the first liposome formulation approved by the FDA, for the treatment of AIDS-related Kaposi’s sarcoma in patient’s refractory or intolerant to combination chemotherapy
Exosome	Using naturally secreted nanoscale vesicles from cells (30–150 nm) as delivery carriers, naturally exhibiting low immunogenicity	Natural targeting, low immunogenicity/toxicity, crossing biological barriers, natural cargo delivery	Drug leakage, low encapsulation efficiency, challenges in large-scale production	No exosome products have been approved for marketing currently. Exosomes products in the clinical stage include exoSTING, exoIL-12 and iExosomes
Cell membrane biomimetic nanoparticles	Encapsulate synthetic nanoparticles (such as PLGA) with cell membranes (red blood cells, cancer cells, immune cells) to give them cellular functions	Immune evasion, homologous targeting functionalization, combination therapy	Extremely low yield, difficult to control drug-loading efficiency, heterogeneity	Erytech has developed a chemotherapy drug delivery platform based on red blood cell encapsulation technology, and its products have entered phase III clinical trials in areas such as acute lymphoblastic leukemia
Hydrogel	Place the drug-loaded hydrogel in the tumor or postoperative cavity through local injection to provide sustained and controlled drug release	Continuous release, High local concentration and low systemic toxicity, responsiveness, filler effect	Invasive administration may cause inflammation and uneven drug distribution	Eligard® is a subcutaneous injection of a PLGA polymer solution that forms a biodegradable *in situ* hydrogel upon contact with body fluids, allowing sustained release of leuprorelin for several months, used for the treatment of advanced prostate cancer
Viral vector	Infect cells with modified viruses (such as adenoviruses or lentiviruses) to deliver genes encoding pro-ICD proteins	Efficient transduction, sustained expression, self-amplification	Immunogenicity, Safety risks, Vector capacity limitations, Production and regulatory complexities	DeltaRex-G is a retroviral vector encoding a cytocidal mutant dominant-negative cyclin G1 gene, exert an anti-tumor effect
Apoptotic vesicles	A type of double-layered lipid structure that encloses cellular contents produced during apoptosis	Low immunogenicity, easy to store, reduces the risk of blood clotting, and can be used for engineering and drug delivery	Heterogeneity, yield, purity, and integrity	No cell apoptotic vesicles vesicle products have been approved for market release

Exosomes, which are naturally secreted nanovesicles ranging from 30 to 150 nm in diameter, function as endogenous delivery systems for intercellular communication (Batista et al., 2024). As carriers for intercellular communication, they exhibit low immunogenicity and a unique ability to penetrate biological barriers. They can carry proteins, nucleic acids, and small-molecule drugs. However, challenges in isolation, purification, and scalable manufacturing have limited their clinical translation. In a promising application, engineered exosomes derived from breast cancer cells were used to co-deliver the ICD inducer ELANE and the TLR3 agonist Hiltonol. This approach promoted *in situ* activation of type 1 conventional dendritic cells (cDC1s), improved CD8^+^ T cell responses and demonstrated potent antitumor activity in both mouse models and human breast cancer organoids (Huang et al., 2022).

Cell membrane-mimicking nanoparticles are engineered by coating synthetic nanoparticle cores with natural cell membranes (e.g., red blood cells, cancer cells, or immune cell membranes), endowing them with complex biological functionalities (Tan H. et al., 2025). However, their fabrication involves technically demanding steps, including membrane extraction and fusion, which pose challenges for standardization and scalable production (Wang Y. et al., 2023). A targeted delivery and regulation system developed based on platelet-neutrophil hybrid membranes was designed for cell-specific responses to the pathophysiological features of wound healing, stimulates dendritic cell maturation and activates T cell immunity for postoperative immunotherapy, preventing tumor recurrence (Sheng et al., 2024).

Hydrogel-based local delivery systems form three-dimensional networks structure through cross-linked polymers that can encapsulate drugs (Chen K. et al., 2024). The drugs are released through diffusion or degradation of the hydrogel, maintaining effective concentrations locally and avoiding frequent administration (Mikhail et al., 2023). However, such systems are generally unsuitable for deep or diffusely metastatic tumors. Viral vectors offer an alternative with exceptionally high transduction efficiency and potential as *in vivo* platforms for tumor vaccine production. But it may trigger strong immune responses and carries risks such as insertional mutagenesis and replication (Huang et al., 2020). The selection of an appropriate delivery strategy should be guided by specific therapeutic requirements, including the need for systemic versus local delivery, the physicochemical properties of the drug, and targeting precision. Looking forward, the integration of multiple strategies to achieve more precise and intelligent ICD-induced therapy.

Apoptotic vesicles (apoVs) are endogenous nanoscale carriers that differ fundamentally from synthetic nanocarriers. They inherently display surface molecules for tissue targeting and are naturally loaded with a diverse cargo of tumor antigens and endogenous immunomodulators, functioning as intrinsically smart vaccine vectors. Critically, the ICD process triggered by many NPs potently stimulates the biogenesis and release of apoVs from dying tumor cells. These ICD-generated apoVs are uniquely loaded with a broad spectrum of tumor-associated antigens and DAMPs (Park et al., 2018). This coupling suggests a novel combinatorial strategy: NPs serve as precise triggers to initiate ICD within tumors, while the subsequently released apoVs as in situ-generated, endogenous carriers that efficiently deliver immunogenic signals to antigen-presenting cells, which leverages the strengths of both targeted immune activation by NPs and superior antigen presentation by natural vesicles.

## 5 Challenges and future perspectives

Currently, natural product drugs that directly use “ICD induction” as the main marker have not yet been officially approved for cancer treatment. However, their clinical translation has a solid foundation. Firstly, many natural products with ICD-inducing activity (such as curcumin, artemisinin derivatives, and baicalin) have been used in traditional medicine for hundreds or even thousands of years. This provides preliminary human experience evidence for their relatively good safety and lower systemic toxicity, which is significantly better than entirely synthetic new chemical entities and may shorten the preclinical toxicity evaluation period (Li et al., 2021). Secondly, some natural products or their derivatives (such as artemisinin derivatives) are already approved drugs (for antimalarial use). Conducting clinical studies on repurposing these drugs can make full use of the known pharmacokinetics and safety data, potentially bypass some preclinical studies and more quickly enter Phase II clinical trials to evaluate their synergistic effects when combined with ICIs. For example, artesunate has been observed to enhance immune cell activity and increase T cell infiltration in the tumor microenvironment in some patients with advanced tumors, which is consistent with the activation mechanism of ICD. Most importantly, the rapid development of cutting-edge technologies, including organoids, single-cell sequencing, and spatial transcriptomics now offers unprecedented resolution to dissect the precise molecular pathways and immune regulatory networks of NPs-induced ICD in models that closely recapitulate human physiology. These tools enable the accurate mapping of the effects of NPs-induced ICD on each cell type within the TME, revealing how ICD reprograms immune cells, stromal cells, and even tumor cell subtypes. Such insights not only help identify novel biomarkers and therapeutic targets but also pave the way for selecting patient populations most likely to respond based on the immune landscape of their tumors.

However, it is undeniable that the clinical benefits of NPs-induced ICD still face a series of formidable challenges. In preclinical models, cell surface CRT or serum HMGB1 can be detected. But in human clinical trials, real-time quantitative monitoring of ICD occurrence is a huge challenge. Due to the lack of standard and practical biomarkers, it is impossible to verify whether a drug truly induces ICD in patients, or to determine the optimal biological dose, leading to blindly designed clinical trials. To address this difficulty, one could consider shifting from static detection to dynamic imaging. Develop radiolabeled or fluorescently labeled antibodies/peptides that can specifically target CRT on the surface of tumor cells. We propose a highly promising strategy: by employing imaging modalities such as positron emission tomography (PET) or optical imaging, this approach allows for real-time, spatial mapping of ICD occurrence, revealing both the specific regions and the extent of immunogenic cell death within tumor tissues. Additionally, construct tumor cells in which a reporter gene (such as luciferase) can be expressed following the activation of key ICD pathways (such as PERK/ATF4/CHOP), allowing dynamic observation of ICD initiation and intensity through bioluminescence imaging. And a multi-indicator combined detection “ICD signal panel” can be established. For example, by combining CRT exposure level, HMGB1 release amount, ATP concentration’ and setting a comprehensive score, it may reflect the occurrence of ICD more specifically and sensitively than any single indicator. Alternatively, instead of directly detecting DAMPs, one could monitor the downstream immune response triggered by ICD. By analyzing changes in T cell receptor (TCR) diversity in the peripheral blood or tumor tissue of patients before and after treatment through high-throughput sequencing, this could serve as an alternative biomarker, which is closer to the ultimate therapeutic effect. These dynamic monitoring strategies can draw upon the mature workflows of gastrointestinal cancer liquid biopsy, which integrate multiple biomarkers such as ctDNA and exosomes. Based on these experiences, we are considering whether it is possible to further develop multi-analyte liquid biopsy panels for monitoring NPs-induced ICD. By simultaneously analyzing circulating DAMPs, cytokine profiles, and immune cell phenotypes, these panels would enable a systematic assessment of treatment responses and remodeling of the tumor microenvironment.

Extracting a single active ingredient from natural products for research aligns with the modern pharmacological approach of single-target studies. However, this method may overlook the essence of NPs “multi-component, multi-target” synergistic effects (Yuan Y. et al., 2025). Therefore, in our view, for systems with extremely complex components, it may be reasonable not to insist on isolating every component, but rather to use chemical analysis to identify a set of “characteristic marker component groups” related to efficacy, along with the content ranges of key marker components. Potential target sites can be predicted through public databases. Then, all these targets are constructed into a protein-protein interaction network and integrated with the significantly altered signaling pathways identified in omics analyses. By using a data-driven approach, a global network diagram of “multi-component, multi-target, multi-pathway” can be drawn. Another major reason why the vast majority of NPs are effective in preclinical studies but ineffective in clinical settings is poor PK properties, which make it impossible or unsafe to achieve the effective concentrations observed *in vitro* (e.g., 10 μM) in the body. Undeniably, pleiotropic bioactive small molecules can mitigate treatment-related damage and modulate inflammation, which is crucial for organ protection. Nanodelivery technology is currently the most important and effective strategy. Its core is to create a dedicated vehicle for natural product molecules, increasing drug solubility and stability, allowing the drug to accumulate in tumor sites, enhance local concentration, and reduce systemic toxicity (Guevara et al., 2021). Without affecting pharmacological activity, decorate natural product molecules to improve their intrinsic properties. Covalently attach the original drug to a temporary transport group (such as a phosphate ester or amino acid) to form a prodrug that is inactive *in vitro*. This prodrug has better water solubility or membrane permeability, and once in the body, it releases the original drug through enzymatic hydrolysis (Ding et al., 2022). This approach can specifically address issues such as first-pass effect, poor water solubility, and low targeting. To overcome the therapeutic bottlenecks of NPs, research must shift from “broad” to “refined” formulation engineering and molecular design. At the same time, future research should focus on leveraging new technologies and carefully designed clinical trials to conduct in-depth mechanistic exploration, translating these promising preclinical findings into tangible patient benefits.

## 6 Conclusion

In summary, this review highlights that the induction of ICD represents a pivotal mechanism by which numerous NPs -derived active components exert anticancer effects. These compounds predominantly elicit ICD primarily through the induction of ER stress and ROS generation, leading to the emission of DAMPs that activate DCs and CTLs. However, systematic biological approaches are still needed to determine how upstream targets differentially coordinate the magnitude, spatial distribution, and timing of ROS/ER stress. To overcome pharmacokinetic limitations and maximize therapeutic efficacy, advanced strategies such as nanotechnology-driven delivery and rational combinations with ICIs are essential. Although standardization and clinical translation remain challenging, combining the intelligence of NPs with modern immunotherapy and nanotechnology possesses extraordinary potential for developing the next-generation of cancer treatments.
